# Navigating the patient experience in primary healthcare clinics in the eastern province of Saudi Arabia: a secondary analysis study

**DOI:** 10.3389/fmed.2025.1607267

**Published:** 2025-06-17

**Authors:** Mishael Alhuseini, Duaa Aljabri, Arwa Althumairi, Quds Al Saffer

**Affiliations:** ^1^Department of Health Information Management and Technology, College of Public Health, Imam Abdulrahman Bin Faisal University, Dammam, Saudi Arabia; ^2^King Fahad Specialist Hospital, Dammam, Saudi Arabia

**Keywords:** primary healthcare, primary healthcare center, patient experience, patient satisfaction, Saudi Arabia

## Abstract

**Background:**

The patient experience is a critical indicator of healthcare quality, particularly in primary healthcare centers (PHCCs). Understanding patients’ perceptions is essential for improving service delivery and achieving patient-centered care. Despite healthcare reforms in Saudi Arabia, research on patient experiences in PHCCs in the Eastern Province remains limited.

**Objective:**

This study aims to analyze patient experience trends in PHCCs within the Eastern Health Cluster of Saudi Arabia, identifying factors influencing satisfaction levels.

**Methods:**

A secondary analysis was conducted using patient experience data from the Ministry of Health’s Press Ganey survey. Data from 81,211 completed surveys collected between 2022 and 2023 were analyzed. The survey assessed seven domains of patient experience, and statistical tests, including *t*-tests and one-way ANOVA, were used to examine variations by demographic factors.

**Results:**

The overall patient experience score was 85.4%, with the highest satisfaction in the registration process (88.6%) and the lowest in the “moving through” domain (79%). Elderly patients (>65 years) reported higher satisfaction compared to younger adults (18–34 years) (*p* < 0.05). Male patients expressed slightly greater satisfaction than females (*p* < 0.05). Satisfaction varied across health networks, with Network C receiving the highest scores (88.8%) and Network B the lowest (83.2%). Patient satisfaction improved from 2022 (84%) to 2023 (86.4%).

**Conclusion:**

The patient experience in PHCCs within the Eastern Health Cluster has improved over time, reflecting ongoing healthcare reforms. However, disparities exist across demographic groups and health networks. Addressing patients’ concerns about wait times and service flows is necessary to enhance satisfaction. Future research should explore qualitative aspects of the patient experience and assess interventions to improve healthcare accessibility and equity.

## 1 Introduction

The landmark 2001 report *Crossing the Quality Chasm*, by the National Academy of Medicine, marked a paradigm shift in healthcare by emphasizing patient-centered care as a cornerstone of quality improvement (1). The report identified six essential dimensions of high-quality healthcare: effective, efficient, equitable, safe, timely, and patient-centered. Patient-centered care was defined as “respectful of and responsive to individual patient preferences, needs, and values, ensuring that patient values guide all clinical decisions” (2). These principles have since guided clinicians, researchers, and policymakers as they integrate patient experience measures as critical indicators for evaluating and improving patient-centered care.

Understanding patients’ perceptions and experiences is fundamental for enhancing the quality of healthcare systems. The Beryl Institute defines the patient experience as “the sum of all interactions, shaped by an organization’s culture, that influence patient perceptions across the continuum of care” (3). Similarly, the Agency for Healthcare Research and Quality emphasizes that the patient experience includes key aspects of healthcare delivery valued by patients, such as timely appointments, accessible information, and effective communication with providers (4). Other studies have further demonstrated that higher levels of patient satisfaction are associated with improved adherence to treatment plans, leading to better health outcomes (5).

Primary healthcare (PHC) is widely recognized as the cornerstone of modern healthcare systems. Providing high-quality PHC that aligns with patients’ expectations is essential for achieving their satisfaction and strengthening health system performance. Previous studies have demonstrated that strong, community-based primary care improves both the responsiveness and overall effectiveness of health systems (6).

In a study involving 34 European countries, nearly three-quarters (74%) of patients who consulted a primary care physician reported an improved ability to manage their health conditions following the consultation (7). The same study also highlighted that a longitudinal, continuous relationship between primary care physicians and their patients was associated with a lower likelihood of emergency department utilization. Further evidence suggests that increased investment in PHC not only enhances patient outcomes but also reduces reliance on secondary care services, contributing to significant cost savings. For example, a 4-year study in the United Kingdom found that greater investment in PHC resulted in a £165 million reduction in secondary care expenditures (8).

Like many countries, Saudi Arabia faces significant healthcare challenges driven by demographic transitions, including an aging population and the growing prevalence of chronic diseases. By 2030, the number of individuals aged 50 and above is projected to nearly double, to reach 25% of the population, compared to 15% in 2020 (8). According to the *Saudi Health Status Statistics 2022* report, the prevalence of chronic disease among adults is almost 20%. Additionally, Saudi Arabia has one of the highest global prevalence rates of diabetes, affecting 15.8% of the population (9). This demographic trend, coupled with chronic disease prevalence, underscores the urgent need for preventive healthcare and patient adherence initiatives.

To address these challenges, Saudi Arabia’s healthcare sector is undergoing transformative changes under the National Transformation Program and Vision 2030 framework. These reforms aim to establish a new healthcare model that improves the population’s health, enhances equity of access, and reduces the burden on secondary and tertiary care (10). To enhance access to healthcare services and streamline patients’ transitions among different levels of care, the Ministry of Health (MOH) introduced the concept of health clusters in all regions of the Kingdom as one of the national transformation projects in healthcare. Each healthcare provider, including the hospitals and primary healthcare centers (PHCCs) within a cluster, is required to coordinate and collaborate to meet the needs of the defined population (11).

Despite these efforts, the use of PHCCs remains inadequate. A national study found that while 84% of respondents were aware of PHC services in their districts, 30% had never made use of them (12). Additionally, a Riyadh-based study revealed that 63% of participants lacked a regular primary care provider, and 44% perceived emergency department services as superior to PHC (13). Most visits to PHCCs were for immunization services or referrals to secondary or tertiary hospitals (12). In response, the MOH has implemented the Patient Experience Measurement Program as part of its national transformation initiative (14). In partnership with Press Ganey, an independent third-party organization, the program uses patient experience surveys to evaluate care across domains such as appointment processes, provider interactions, and overall satisfaction. These surveys aim to enhance transparency, empower patients, and encourage providers to improve service quality.

PHC plays an essential role in the world’s healthcare systems as it targets individuals, families, and communities and provides a range of preventive and curative healthcare services (6). Understanding patients’ perceptions and experiences is a core element for improving the quality of care in any PHC system. An excellent patient experience increases patient engagement and adherence to providers’ instructions, clinical processes, and health outcomes (15). While the patient experience in PHCCs has been explored in various regions of Saudi Arabia, limited research has focused on public PHCCs in the Eastern region. This study aims to fill this gap by analyzing patients’ experiences in MOH-run PHCCs in the Eastern Province for the years 2022 and 2023. The findings aim to provide actionable insights for improving healthcare quality, advancing PHC services, and aligning healthcare systems with patients’ needs.

## 2 Materials and methods

### 2.1 Study design and settings

This study used secondary data obtained retrospectively from the MOH and derived from the Press Ganey Patient Experience Survey, which is responsible for collecting patient experience data from across Saudi Arabia. The Press Ganey survey is extensively validated in the literature and has been implemented in more than 35,000 healthcare facilities worldwide (16). For this study, the survey was translated into Arabic and validated following the World Health Organization’s guidelines for the translation and adaptation of instruments (14).

**FIGURE 1:**
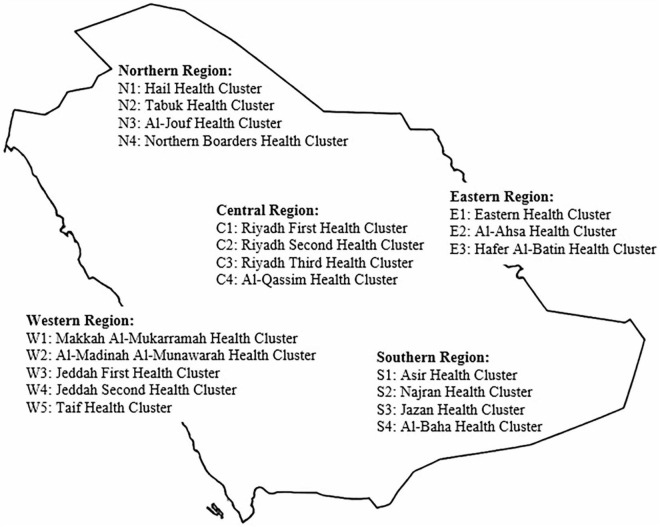
Distribution of healthcare clusters in Saudi Arabia.

The data for this study were collected from urban PHCCs within the Eastern Health Cluster. As of 2022, Saudi Arabia was organized into 20 health clusters distributed across five main regions (Figure 1). In the Eastern Region, three health clusters were established: the Eastern Health Cluster, the Al-Ahsa Health Cluster, and the Hafer Al-Batin Health Cluster (17). The Eastern Health Cluster provides healthcare services to more than two million beneficiaries through 120 primary care centers; a medical city, a tertiary-level medical complex that serves as a central hub for specialized and high-complexity care within the region; and 22 general and specialty hospitals (18). The cluster is divided into multiple health networks based on geographic distribution. This study focused on 60 PHCCs located in urban areas and organized into three health networks (A, B, and C).

### 2.2 Participants: sample size and data collection techniques

The study participants comprised patients who voluntarily responded to the MOH Patient Experience survey online after providing consent to participate. The survey was distributed to participants, or to the parent or legal guardian of those under the age of 18, via text messages to their mobile phones following each visit to an MOH PHC. A randomly selected subset of patients seen each day received invitations to complete the survey (19). Patients were able to access the survey for up to 14 days post-visit. The data covered the period from January 1, 2022, through December 31, 2023.

### 2.3 Variables

The patient experience survey comprises a set of questions about the patient’s experiences during their last PHC visit. The survey tool consists of 22 questions divided into seven domains: registration (two questions), appointment (two questions), moving through your visit (three questions), experiences with nurses (three questions) and care providers (four questions), personal issues (four questions), and patients’ perceptions of the practice (four questions). The total score is calculated from the mean scores from each of these seven domains using proprietary equations (Table 1). Each question measures responses on a Likert scale ranging from 1 (indicating not satisfied at all) to 5 (indicating extremely satisfied), which are converted to a 0–100 scale. Higher total scores indicate a better patient experience with the clinical encounter.

**TABLE 1:** The seven domains and related questions of the questionnaire.

Domain	Question
Registration	1. Ease of registration upon arrival
	2. Courtesy of the receptionists
Appointments	3. Ease of scheduling your appointment
	4. Ease of contacting the center (e.g., email, phone, web portal / application)
Moving through	5. Wait time at clinic (from arriving to leaving)
	6. Degree to which you were informed about any delays
	7. Comfort of the waiting area
Nurse	8. Friendliness/courtesy of the nurse
	9. Concern the nurse showed for your problem
	10. How well the nurse listened to you
Care provider	11. Explanations the physician gave you about your problem or condition
	12. Concern the physician showed for your questions or worries
	13. Physician’s efforts to include you in decisions about your care
	14. Physician’s discussion of any proposed treatment (options, risks, benefits, etc.)
Personal issues	15. Availability of parking
	16. How well the staff protected your safety (by washing hands, wearing ID, etc.)
	17. Our concern for your privacy
	18. Center Cleanliness
Patient perception	19. Likelihood of your recommending this physician to others
	20. How well the staff worked together to care for you
	21. Likelihood of your recommending this center to others
	22. How satisfied are you with the healthcare experience at the Primary Care Centers?

### 2.4 Statistical methods

Surveys marked as incomplete were excluded, and only those marked as submitted were included in the analysis. As answering all survey questions as not mandatory, some submitted surveys contained missing responses. Based on the literature, missing data that are completely at random and account for less than 5% of the dataset are generally considered negligible, with simple imputation techniques, such as mean imputation, deemed appropriate (20). In this study, the proportion of missing responses for each survey question was calculated and found to be below 5%. Consequently, the mean score for each question was imputed for the missing responses.

Descriptive statistics were generated for the demographic variables gender (male, female), nationality (Saudi, non-Saudi), year (2022, 2023), and network (A, B, or C). A frequency analysis was performed for age, which was further categorized into one of five groups: (0–17, 18–34, 35–49, 50–64, and ≥ 65 years). Age categorizations were based on both scientific reasoning and practical considerations for healthcare surveys (21). Parametric tests were used for inferential statistical analysis. According to the central limit theorem (CLT), with a sufficiently large sample size (usually *n* > 30), parametric tests are appropriate because the distribution of the sample mean will approximate a normal distribution. The CLT states that, as the sample size increases, the sampling distribution becomes more normal, even if the original data are not. Additionally, the variability of the sampling distribution decreases as the sample size grows, and the mean of the sampling distribution is equal to the mean of the population from which it was drawn (22).

The mean and standard deviation (SD) of the satisfaction scores were calculated for the seven domains. Furthermore, to compare the means of satisfaction among networks and age groups, we applied a one-way ANOVA test, making it possible to compare the means across multiple groups and to ascertain whether there were any significant differences in the mean values of response variables for different categories. A *t*-test was used to compare the means of satisfaction between the gender categories (female vs. male) and years (2022 vs. 2023). A *p*-value of less than 0.05 was considered the threshold for statistical significance in all analyses.

The collected data were transferred to a Microsoft Excel file and the data were analyzed statistically using IBM SPSS Statistics for Windows, Version 29.0.

## 3 Results

**FIGURE 2:**
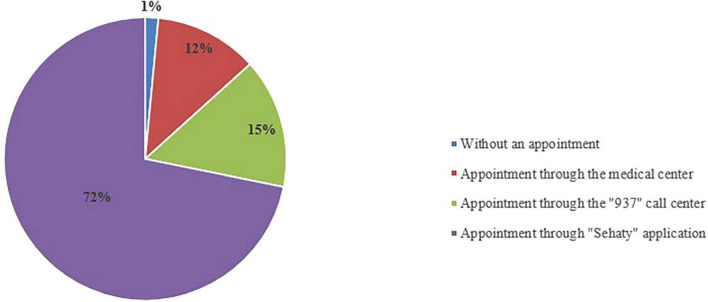
How did you attend the PHC?

A total of 106,168 responses from patients who visited the selected PHCCs received the survey; however, only 81,211 participants (76.5%) completed the patient experience survey. Most of the participants (72%) booked their appointment via the Sehaty application, an MOH unified platform that allows users to access health services, as shown in Figure 2. An additional 15% of the respondents booked their PHC appointment via the 937 MOH Call Center (23).

Table 2 displays the demographic characteristics of the study participants. Their mean age was 30.1 years (SD ± 20.9), though a plurality of them were under 18 years of age (33.4%), making this the largest age cohort in the study. Among the participants, 52% were female and 48% were male; 94% were Saudi, while 6% were non-Saudi. Regarding the geographic distribution of the study participants, most of the survey respondents were from Network B (44.4%), followed by Network C (33.3%) and Network A (22.3%).

**TABLE 2:** The descriptive statistics of survey responses (*n* = 81,211).

Variable	Categories	Frequency (N)	%
Age*	0–17	27,124	33.4%
	18–34	16,513	20.3%
	35–49	20,295	25%
	50–64	14,341	17.7%
	≥ 65	2,928	3.6%
Gender	Female	42,190	52%
	Male	39,009	48%
Nationality	Saudi	76,271	94%
	Non-Saudi	4,940	6%
Year	2022	36,019	44.4%
	2023	45,192	55.6%
Network	A	18,131	22.3%
	B	36,019	44.4%
	C	27,061	33.3%

*Age: Median = 32, Mean = 30.16, SD ± 20.96

The overall results of the patient experience scores across the various domains are presented in Table 3. The overall patient experience score was approximately 4.27 out of 5 (85.4%, SD ± 0.85). Among the domains, the “registration” process received the highest mean satisfaction score, 4.43 (88.6%, SD ± 0.96), indicating that more people were confident in and satisfied with this aspect of the service. In contrast, the “moving through” domain recorded the lowest satisfaction score, with an overall satisfaction rate of 79%.

**TABLE 3:** The overall results of the patient experience scores of the seven domains of the Press Ganey survey in PHC settings (*n* = 81,211).

Domain	Mean	% out of 5	SD
Registration	4.43	88.6%	±0.96
Appointment	4.19	83.8%	±0.96
Moving through	3.95	79.0%	±1.16
Nurse	4.35	87.0%	±1.01
Care provider	4.38	87.6%	±1.09
Personal issues	4.27	85.4%	±0.87
Patient perception	4.03	80.6%	±1.09
Overall experience score	4.27	85.4%	±0.85

Patients’ overall experience scores varied across age groups, with those aged over 65 reporting a better experience with PHC services compared to those aged 18–34 (Table 4). Further analyses of age-related differences across the patient experience domains revealed an intriguing interaction effect: Most age groups reported the lowest satisfaction with the “moving through” domain, but rated the “registration” services highly. All comparisons were statistically significant (*p* < 0.05).

**TABLE 4:** Comparison of experience scores per age (one-way ANOVA test).

Domain	Age					*F*	*P*-value
	**0–17** ***n* = 27,124**	**18–34** ***n* = 16,513**	**35–49** ***n* = 20,295**	**50–64** ***n* = 14,341**	**>65** ***n* = 2,928**		
	**Mean ± SD**	**Mean ± SD**	**Mean ± SD**	**Mean ± SD**	**Mean ± SD**		
Registration	4.40 ± 1.00	4.26 ± 1.09	4.49 ± 0.90	4.55 ± 0.80	4.60 ± 0.71	229.1	<0.001
Appointment	4.23 ± 0.95	4.07 ± 1.03	4.16 ± 0.99	4.26 ± 0.89	4.35 ± 0.78	114.8	<0.001
Moving Through	3.93 ± 1.20	3.70 ± 1.28	4.03 ± 1.11	4.11 ± 0.99	4.23 ± 0.89	330.4	<0.001
Nurse	4.31 ± 1.06	4.18 ± 1.17	4.42 ± 0.93	4.47 ± 0.82	4.53 ± 0.72	230.3	<0.001
Care Provider	4.34 ± 1.10	4.14 ± 1.30	4.46 ± 1.03	4.58 ± 0.84	4.64 ± 0.72	407.1	<0.001
Personal Issues	4.24 ± 0.90	4.14 ± 0.97	4.34 ± 0.82	4.36 ± 0.75	4.39 ± 0.71	189.1	<0.001
Patient perception	4.26 ± 1.13	4.01 ± 1.27	4.38 ± 1.01	4.51 ± 0.83	4.59 ± 0.73	533.2	<0.001
Overall experience score	4.24 ± 0.88	4.07 ± 0.96	4.33 ± 0.79	4.41 ± 0.70	4.48 ± 0.63	393.6	<0.001

Regarding gender differences, male participants reported slightly higher satisfaction levels than female participants (86% vs. 84.6%), as shown in Table 5. Among the patient experience domains, female participants expressed the lowest satisfaction with the “moving through” domain, compared to their male counterparts. Furthermore, the level of satisfaction among survey participants was different between the years 2022 and 2023, with respondents reporting higher levels of satisfaction in 2023 compared to 2022, as presented in Table 6. All comparisons were statistically significant (*p* < 0.05). In terms of the patients’ mean satisfaction scores by region, the highest score was seen in Network C (4.44; 88.8%), while the lowest patient experience score was in Network B (4.16; 83.2%), as shown in Table 7.

**TABLE 5:** Comparison of experience scores according to the participant’s gender (*t*-test).

Domain	Gender		*t*	*P*-Value
	**Female *n* = 42,190**	**Male *n* = 39,009**		
	**Mean ± SD**	**Mean ± SD**		
Registration	4.41 ± 0.97	4.44 ± 0.95	−4.91	<0.001
Appointment	4.15 ± 0.98	4.24 ± 0.94	−12.49	<0.001
Moving through	3.90 ± 1.18	4.00 ± 1.14	−12.67	<0.001
Nurse	4.32 ± 1.02	4.38 ± 0.99	−8.74	<0.001
Care provider	4.34 ± 1.11	4.43 ± 1.06	−11.07	<0.001
Personal issues	4.25 ± 0.88	4.29 ± 0.85	−7.17	<0.001
Patient perception	4.25 ± 1.11	4.35 ± 1.06	−13.45	<0.001
Overall experience score	4.23 ± 0.86	4.30 ± 0.83	−12.33	<0.001

**TABLE 6:** Comparison of experience scores according to the survey’s year (*t*-test).

Domain	Year		*t*	*P*-value
	**2022** ***n* = 36,019**	**2023** ***n* = 45,192**		
	**Mean ± SD**	**Mean ± SD**		
Registration	4.38 ± 0.99	4.46 ± 0.94	-12.21	<0.001
Appointment	4.15 ± 0.98	4.22 ± 0.95	-10.64	<0.001
Moving through	3.85 ± 1.19	4.03 ± 1.13	-21.99	<0.001
Nurse	4.31 ± 1.09	4.38 ± 0.94	-8.32	<0.001
Care provider	4.32 ± 1.12	4.43 ± 1.06	-13.48	<0.001
Personal issues	4.20 ± 0.90	4.32 ± 0.83	-19.32	<0.001
Patient perception	4.21 ± 1.11	4.37 ± 1.06	-20.90	<0.001
Overall experience score	4.20 ± 0.88	4.32 ± 0.81	-18.52	<0.001

**TABLE 7:** Comparison of experience scores per network (one-way ANOVA test).

Domain	Network			*F*	*P*-value
	**Network A *n* = 18,131**	**Network B *n* = 36,019**	**Network C *n* = 27,061**		
	**Mean± SD**	**Mean± SD**	**Mean± SD**		
Registration	4.36 ± 1.00	4.34 ± 1.02	4.59 ± 0.82	568.6	<0.001
Appointment	4.23 ± 0.94	4.07 ± 1.05	4.33 ± 0.84	568.5	<0.001
Moving through	3.88 ± 1.19	3.86 ± 1.21	4.12 ± 1.05	450.7	<0.001
Nurse	4.27 ± 1.06	4.24 ± 1.08	4.54 ± 0.84	775.5	<0.001
Care provider	4.30 ± 1.14	4.28 ± 1.16	4.57 ± 0.91	621.0	<0.001
Personal issues	4.24 ± 0.89	4.16 ± 0.93	4.43 ± 0.74	728.2	<0.001
Patient perception	4.21 ± 1.13	4.18 ± 1.16	4.51 ± 0.90	798.3	<0.001
Overall experience score	4.21 ± 0.87	4.16 ± 0.90	4.44 ± 0.71	901.9	<0.001

## 4 Discussion

This study has shown that patient experience survey results have exhibited a positive trend over 2 years in Saudi Arabia. Previous studies in Saudi Arabia have reported that patient satisfaction has been increasing annually, with rates rising from 71.7% in March 2018 to 75.1% in March 2019, based on quarterly sample surveys conducted in PHCCs by Press Ganey (24). Our findings align with the literature, as the overall satisfaction rate with PHCs’ services increased from 84% in 2022 to 86.4% in 2023. These results are comparable to studies conducted in Bahrain and Qatar, which reported that approximately 75–80% of patients visiting PHCCs in those countries were generally satisfied (25).

The improvement in PHC patient experience scores can be attributed to recent health reforms in Saudi Arabia, which are devoted to enhancing the patient experience, improving health outcomes, and reducing costs by promoting a patient-centered approach in PHC settings (10). Moreover, by mid-2019, these reforms had increased not only PHC visits, patient satisfaction, and rural community coverage, but also the rate of screening for prevalent chronic diseases (24). Therefore, the findings of our study appear logical, given the significant efforts made by the Saudi MOH to enhance healthcare providers’ professional skills through training programs, continuous medical education, and improved competency in PHC practices and services (26). According to the latest MOH patient experience reports, the overall score has exceeded the Gulf Cooperation Council (GCC) benchmark score of 78.46%. However, the score still falls short of the international benchmark of 92.89% (14).

The results of this study demonstrate that elderly patients (> 65 years) are the most satisfied with PHC services. These results are similar to a review that suggested a positive association between age and the patient experience, with experience ratings tending to increase with a patient’s age. The review reported that patients aged 65 and above generally report better experiences with their care, compared to younger cohorts in several PHCCs in the United States, South Africa, and the United Kingdom (27). Interestingly, despite the general trend showing increasing satisfaction with advancing age, our results contradict a study that was conducted among PHC patients in Saudi Arabia, in which younger patients, those in the 18–30 age group, reported higher levels of satisfaction compared to older patients (28).

Although limited research exists on the causes of age-related differences in patients’ experiences, prior studies have suggested that physicians are more likely to engage in patient-centered interactions with older individuals compared to younger ones (27). Consistent with these findings, our study showed that the care provider domain received the highest experience scores among older individuals, compared to other domains. Similarly, a study conducted in GCC countries reported that older populations were primarily satisfied with physicians’ attitudes (29). In contrast, the “moving through” domain, which assesses wait times, delays, and the comfort of the waiting area, received the lowest scores among young adults (18–34 years), compared to the other domains.

Previous research in the service industry shows that different generations have distinct preferences when it comes to employee interactions. Older adults (aged 50 and above) often value qualities such as empathy, expertise, and effective problem-solving, while younger people (aged 25 and under) prioritize friendliness, attentiveness, and quick service (30). These generational differences provide valuable insights and highlight the need for further exploration in future studies.

In addition to the above, our study results showed that female participants were less satisfied than male patients were. In contrast, an analysis of the relationship between patient characteristics and overall satisfaction with PHC services in Riyadh found that female participants reported higher levels of satisfaction compared to males (28). Moreover, research assessing the influence of a patient’s gender on their satisfaction with health services in family medical practices revealed no significant difference between men and women in their ratings of various aspects of primary care visits. This study suggested that the absence of differences might be due to cultural, religious, and/or social factors in Saudi Arabia (31). However, the differences observed in these study results might be attributed to the fact that men and women have significantly different patient service needs.

Gender-related disparities are also evident for specific components of healthcare services: females frequently raise concerns related to nursing care, whereas males tend to express dissatisfaction with doctor–patient interactions and wait times (32). By contrast, some studies suggest that gender does not significantly affect overall patient experience ratings. These findings emphasize the complexity of gender’s influence on healthcare experiences, indicating that it should not be regarded as a singular or definitive predictor of patient satisfaction (27).

In the present study, overall patient experience scores varied across the three selected health networks within the Eastern Health Cluster. Specifically, the highest satisfaction level was observed in Network C, followed by Network A, with the lowest level reported in Network B. This disparity may be attributed to differences in the distribution of PHCCs and population density among the three health networks. According to the Saudi General Authority for Statistics, Network A operates 10 PHCCs, serving approximately 658,550 residents; Network B has 22 PHCCs, serving over 1,500,000 residents; and Network C includes 26 PHCCs, catering to 552,442 individuals (33).

The unequal distribution of PHCCs and health workers poses a significant challenge, with these networks appearing to be heavily burdened due to an imbalanced ratio of PHCCs to population. The regional variations of PHCCs justify the differences in patient satisfaction scores among the three health networks. In addition, a national study conducted in Saudi Arabia reported that approximately 56% of PHCCs are located in rural areas. Notably, rural regions tend to have a higher number of PHCCs, compared to urban areas, which can be explained by the very small size of the rural population in Saudi Arabia (only 17% of the population lives in a rural area) (10).

## 5 Limitations

While the use of a third party (Press Ganey) improved data quality and minimized collection bias, not all PHC recipients had an equal opportunity to participate. Although the survey achieved a relatively high response rate (76.5%), response bias may still be present, as dissatisfied patients could have been less likely to complete the survey.

The observed increase in satisfaction between 2022 and 2023 may also have been influenced by factors beyond health system reforms, including the post-pandemic normalization of healthcare access. Enhancements introduced by the MOH in response to COVID-19 may also have contributed to these improvements.

Recall bias is a common limitation in patient-reported experience measures, as responses may be influenced by memory, mood, or external discussions. While quantitative methods offer broad insights, integrating qualitative approaches could provide deeper context.

These findings reflect urban populations within the Eastern Health Cluster and may not be generalizable to rural areas, where healthcare access and expectations differ. Finally, while differences across years, networks, and demographics were statistically significant, effect sizes were modest. Nonetheless, such patterns may still indicate structural inequities and warrant further exploration.

## 6 Conclusion

In summary, this study highlights several key factors influencing patients’ experiences with PHC services in Saudi Arabia. The overall improvement in patients’ satisfaction in recent years aligns with national health reforms aimed at enhancing primary healthcare quality through patient-centered approaches. However, disparities in satisfaction scores were observed across different health networks, likely due to variations in PHCC distribution and population density. Additionally, demographic factors such as gender and age emerged as significant determinants of the patient experience. While elderly patients (>65 years) reported the highest satisfaction levels, younger adults (18–34 years) were less satisfied, particularly in the domains related to wait times and comfort. Gender-related differences in satisfaction were also evident, with male and female patients expressing distinct concerns, underscoring the complexity of gender’s role in shaping perceptions of healthcare. These findings suggest the need for tailored interventions addressing the unique needs of diverse patient groups and highlight the importance of equitable resource allocation to further improve PHC services. Although Press Ganey surveys offer a standardized patient experience measurement tool, integrating qualitative research methods, such as analyzing free-text comments, conducting interviews, and organizing focus groups, could provide deeper, more nuanced insights. Future studies should investigate the root causes of these disparities and assess approaches for enhancing the delivery of patient-centered care.

## Data Availability

The data analyzed in this study is subject to the following licenses/restrictions: The data supporting the findings of this study are not publicly available due to licensing agreements. Access to the data is restricted to comply with Saudi Ministry of Health requirements. Requests for data access may be considered on a case-by-case basis and subject to appropriate approvals; however, the authors are unable to share the data openly. Requests to access these datasets should be directed to MOH, GDRS-IRB@moh.gov.sa.
